# Platelet-rich fibrin improves repair and regeneration of damaged endometrium in rats

**DOI:** 10.3389/fendo.2023.1154958

**Published:** 2023-08-08

**Authors:** Lele Mao, XiaoXue Wang, Yu Sun, Mukun Yang, Xing Chen, Lei Cui, Wenpei Bai

**Affiliations:** ^1^ Department of Obstetrics and Gynecology, Ninth Clinical Medical College, Peking University, Beijing Shijitan Hospital, Beijing, China; ^2^ Department of Obstetrics and Gynecology, Beijing Shijitan Hospital, Capital Medical University, Beijing, China; ^3^ Department of Plastic Surgery, Beijing Shijitan Hospital, Capital Medical University, Beijing, China

**Keywords:** intrauterine adhesion, uterine infertility, endometrial repair and regeneration, platelet-rich fibrin, gynecologic endocrinology

## Abstract

**Purpose:**

Intrauterine adhesion (IUA) is the most common cause of uterine infertility. This study aims to evaluate whether platelet-rich fibrin (PRF) treatment can stimulate damaged endometrium regeneration in rats.

**Methods:**

First, hematoxylin and eosin (HE) staining, scanning and transmission electron microscopy, and ELISAs were used to evaluate the microstructure of PRF. Then, mechanical damage was used to establish an IUA rat model. A total of 40 SD female rats were randomized to three groups: PRF transplantation group, IUA group, and sham group. Rats were sacrificed at 3, 7, and 14 days and uteruses were obtained for further analysis. Finally, functional and histological recovery of the damaged endometrium was analyzed by pregnancy test, HE staining, Masson’s staining, and immunohistochemistry.

**Results:**

PRF has two distinct zones, platelets and fibrin zone. Long and narrow fibrin fibers interconnected with each other and formed a three-dimensional, flexible, and elastic structure; platelet aggregates were trapped in fibrin fibers, and each platelet is associated with several fibrin fibers. PRF exudates promoted endometrial stromal cell proliferation and migration *in vitro*. PRF transplantation was beneficial for maintaining uterine structure, promoting endometrial luminal epithelium and endometrial gland regeneration, and decreasing fibrotic areas *in vivo*.

**Conclusion:**

Intrauterine administration of PRF was demonstrated to be effective in preventing IUA and stimulating damaged endometrium regeneration in rats. This study not only provided a promising method for its potential in endometrial regeneration in women who suffer from uterine infertility but also may prevent IUA after intrauterine surgery in clinical cases.

## 1 Introduction

Uterine endometrium is essential for embryo implantation and the maintenance of pregnancy, the dysfunction of which can lead to infertility or obstetric complications. Intrauterine adhesion (IUA), also known as Asherman’s syndrome, is the most common cause of uterine infertility. Approximately 43% of infertile women suffer from this disease (1). IUA is characterized by fibrosis within the uterine cavity due to damage to the basal layer of the endometrium, leading to a partial or complete obliteration in uterine cavity (2). Transcervical resection of adhesion (TCRA), followed by adjuvant postoperative treatment, is widely regarded as the first-line treatment strategy for IUA (3). However, the incidence of recurrent IUA was reported to be as high as 62.5% in severe IUA (4). Although estrogen has been suggested as a perioperative adjuvant therapy for preventing recurrent adhesion, the dosage, route of administration, and duration of estrogen therapy are still controversial (3). Attempts to regenerate endometrium have been shown to be effective in preventing recurrent IUA and prompting pregnancy outcomes (5) with the application of mesenchymal stem cells (6–8), while expansion of stem cells is costly and time-consuming.

Platelet-rich fibrin (PRF) belongs to platelet concentrates, in which platelet content is four to five times higher than that in normal blood (9). PRF is produced by short centrifugation of whole blood without the use of anticoagulant or bovine platelet activators. There is a rich source of growth factors in PRF, including transforming growth factor-beta 1 (TGF-β1), platelet-derived growth factor (PDGF), epidermal growth factor (EGF), fibroblast growth factor (FGF), and vascular endothelial growth factor (VEGF), which have been reported to facilitate angiogenesis and endometrial cell proliferation in damaged endometrium (10). For decades, PRF has been applied mainly in oral surgery such as sinus lifting, alveolar bone defect installation, and maxillofacial defect reconstruction (7). The anti-fibrosis effect of PRF has been documented in preventing keloid scar formation (8). However, there is no study evaluating the effect of PRF on endometrial regeneration.

In the present study, PRF was generated and delivered to cover surgical wounds of endometrium in a rat model. Intrauterine fibrosis and endometrium regeneration were evaluated at 3, 7, and 14 days post-surgery. We found that intrauterine administration of PRF was effective in preventing IUA and stimulating damaged endometrium regeneration in rats.

## 2 Materials and methods

### 2.1 Animals

Adult female Sprague–Dawley (SD) rats (Beijing Vital River Laboratory Animal Technology Co., Ltd.) aged 8–10 weeks were used in all experiments in this study. All experimental protocols were approved by the Ethics Committee of Peking University Ninth School of Clinical Medicine, Beijing Shijitan Hospital.

### 2.2 Preparation of PRF

The protocol by Choukroun et al. (9) was used for PRF preparation. Five milliliters of blood harvested from abdominal aorta was collected in 10-ml tubes without adding anticoagulant, followed by immediate centrifugation at 400 g for 10 min. A fibrin clot obtained as the middle layer of centrifuged blood was defined as PRF, which was just between the red corpuscles at the bottom and acellular plasma at the top.

### 2.3 Quantification of growth factors released from PRFe

Freshly generated PRF was maintained within 5 mL of Dulbecco’s Modified Eagle medium Nutrient Mixture F-12 (DMEM/F12, Gibco, CA, USA) and incubated at 37°C in a humidified atmosphere of 5% CO_2_. After 1, 3, 7, and 14 days, culture medium was collected and stored at −80°C, which was replaced with 5 mL of fresh DMEM/F12. The medium collected at each time point was accumulated and was defined as the “100% PRF exudates (PRFe)”. By dilution with fresh DMEM/F12, a 20% PRFe was used in subsequent studies.

The amount of released growth factors in PRFe, including EGF, TGF-α, IGF-I, PDGF-BB, and VEGF-A, at desired time points was quantified using ELISAs (EIAab, Wuhan, China) according to the manufacturer’s instructions. Absorbance was measured at 450 nm using a DTX880 microplate reader (Beckman Coulter, Brea, CA, USA). All samples were measured in duplicate, and three independent experiments were performed for each platelet concentrate at each time point.

### 2.4 Isolation, culture, and characterization of rat endometrial stromal cells

Endometrial stromal cells (ESCs) were isolated from uterine horns of 8- to 10-week-old female SD rats in the estrus phase according to previously described methods (11). Briefly, the tissue fragments were incubated in a 1:1 mixture of 0.25% trypsin and 1 mg/ml dispase (Gibco, USA) medium for 60 min at 4°C, followed by digestion for 45 min at 23°C, and 15 min at 37°C. Trypsin activity was inhibited by adding ESC medium [DMEM/F12 containing 10% FBS and 1% penicillin–streptomycin (PS), Gibco, USA]. After rinsing three times with Hanks’ Balanced Salt Solution (HBBS), samples were subject to digestion with 1 mg/ml collagenase at 37°C for 30 min. Stromal cells were collected by passing the cell suspensions through a 40-μm nylon mesh, followed by centrifugation at 500 g for 7 min. Cell pellet was resuspended in ESC medium and plated at the desired density for passaging.

Immunocytochemical staining was performed to characterize ESCs by incubating cells with monoclonal mouse anti-vimentin antibody (1:1,000, ab8978, abcam) and monoclonal rabbit anti-Cytokeratin18 antibody (1:300, ab133263, abcam), respectively. TRITC-conjugated anti-mouse IgG (goat, 1:50, abcam) and FITC-conjugated anti-rabbit IgG (goat, 1:50, abcam) were used as secondary antibody as indicated.

### 2.5 Cell proliferation and wound-healing assays

For proliferation assay, ESCs were seeded at a density of 1,000 cells/well in 96-well plates with or without 20% PRFe. After removal of ESC medium, cells were incubated with 10% CCK-8 (Dojindo, Kumamoto, Japan) for 2 h. Absorbance was measured at 450 nm on a microplate reader. All samples were assayed in triplicate.

A wound-healing assay was performed to analyze cellular migration *in vitro*. Briefly, ESCs were seeded into a six-well plate and allowed to grow to 90% confluence in complete medium. Cell monolayers were wounded by a plastic tip (1 mm), washed twice to remove cell debris, and incubated with or without 20% PRFe. Cell migration into the wound surface was monitored by microscopy at desired time points. The migration distances were measured as that between the leading edge and wounded line and migration rate was calculated.

### 2.6 Rat IUA model and PRF transplantation

**Figure 1:**
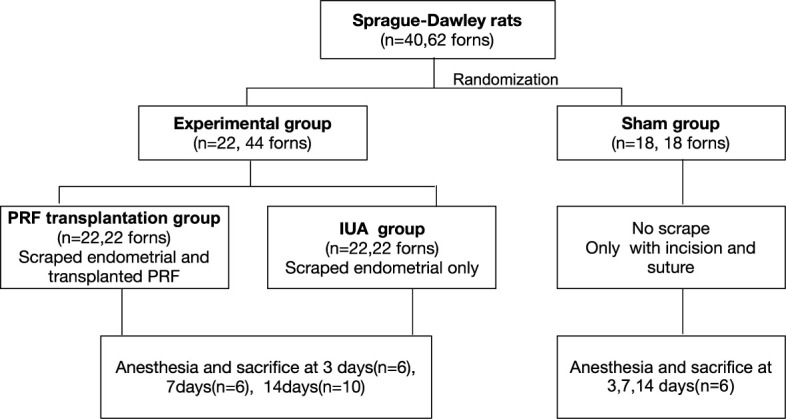
Operation diagram of rat experiments.

An IUA model was established by the mechanical damage method in rats as previously reported (12). Under anesthesia, the uterus was cut lengthwise from the cervix to the fallopian tube to expose. The inner uterine surface was scraped with a No. 21 surgical scalpel blade. The endometrium was completely removed deep in the stroma layer. After washing with saline and exposure to air for 1 h, wounds either received PRF transplantation (PRF group) or were left open (IUA group). Animals in the sham group underwent incision and suturing without uterine curettage. After the surgery, wounds of the uterine and abdomen were closed. Six rats were sacrificed in each group at 3, 7, and 14 days (**Figure 1**).

### 2.7 Histological analysis

The specimens were fixed with 4% paraformaldehyde, embedded in paraffin, and sliced into 5-µm-thick sections for hematoxylin and eosin and Masson’s trichrome staining, respectively. The number of glands and the amount of fibrosis were analyzed by quantitative image-processing software (Image-Pro Plus version 6.0, Media Cybernetics). For immunostaining, rabbit anti-Cytokeratin18 antibody (1:100, ab133263, abcam) or rabbit anti-Ki-67 antibody (1:1,000, ab15580, abcam) were incubated overnight at 4°C, and FITC-conjugated anti-rabbit IgG (goat,1:50, abcam) was incubated as secondary antibody. Dual IF staining of Cytokeratin18 and vimentin was used to demonstrate regenerated endometrium. All samples were counterstained with mounting medium with 4,6-diamidino-2-phenylindole (DAPI, ZLI-9557, ZSGB-BIO, China). Multispectrum fluorescence images were acquired using a confocal microscope (A1, Nikon, Japan). Four fields in each image were selected for counting. Percentages of the positive staining area were quantified using the Image-Pro Plus software.

### 2.8 Scanning electron microscopy and transmission electron microscopy

Scanning electron microscopy (SEM) and transmission electron microscopy (TEM) were performed according to experimental procedures. For SEM, PRF was fixed in 1% glutaraldehyde for 24 h at 4°C and then treated with 1% osmium tetroxide (OsO_4_) for 2 h and dehydrated with graded ethanol. Dehydrated specimens were gold sputter-coated before observing. The microstructure was observed under the scanning electron microscope (S-3400N, Hitachi, Japan). For TEM, PRF was fixed in 2.5% glutaraldehyde, post-fixed in 1% osmium tetroxide, dehydrated in an ascending ethanol series, and embedded in Epon using Beem capsules (Germany). Ultrathin sections were cut and stained with lead citrate and uranyl acetate. The samples were observed under a transmission electron microscope (JEM 2100, Japan).

### 2.9 Statistical analysis

All data were analyzed by SPSS 20.0. Data were tested for normality and presented as mean ± standard deviation (SD). For comparisons between two groups at the same time point, the Student’s *t*-test was used, and for comparisons between four different time points of growth factors, the Kruskal–Wallis test was used. The counting data were analyzed by *χ* ^2^. *p*-values less than 0.05 were considered as statistically significant.

## 3 Results

### 3.1 PRF acts as a possible scaffold and a reservoir of growth factors for endometrial regeneration

**Figure 2:**
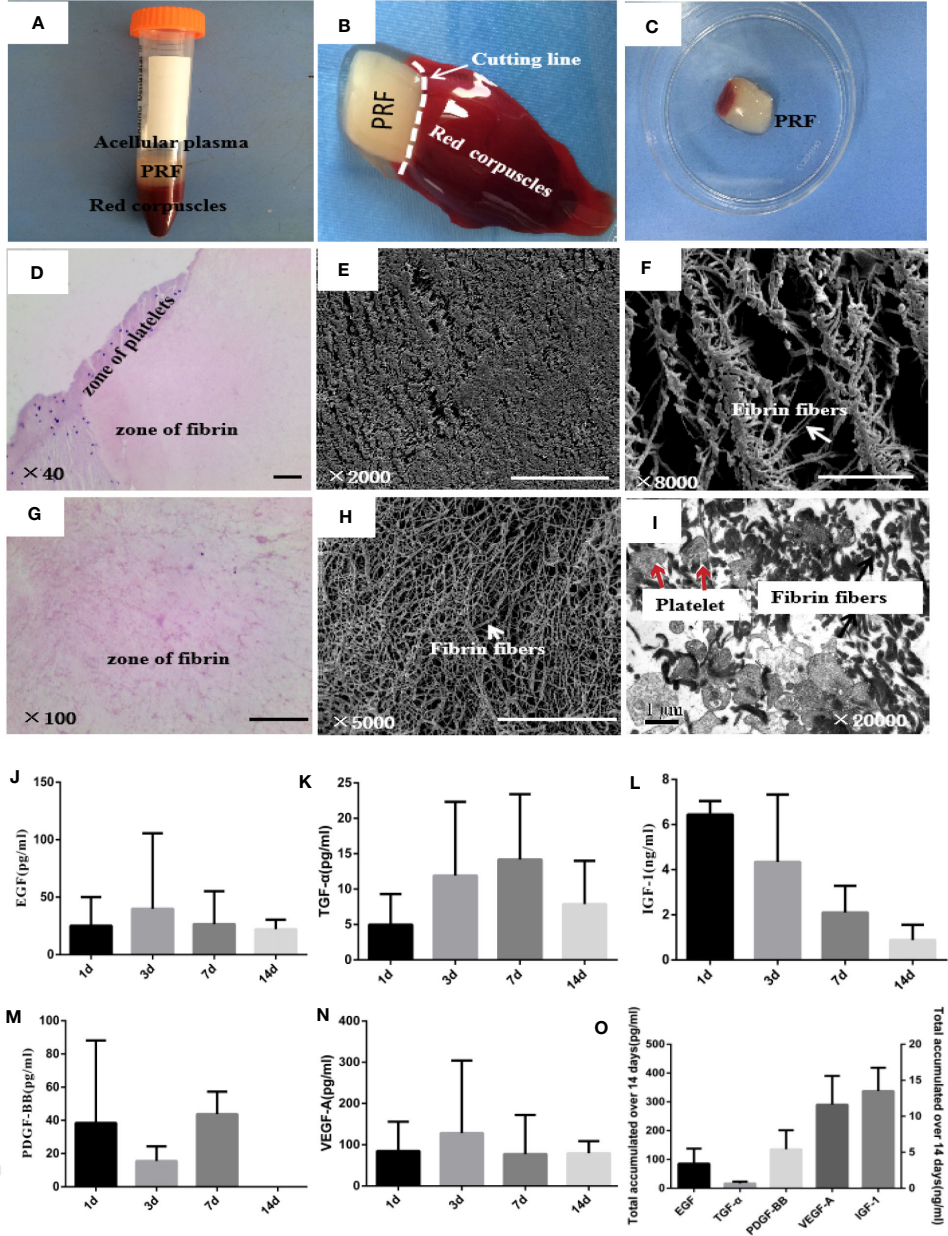
Microstructure and biological characteristics of PRF. **(A)** Blood sample was separated into three layers after centrifugation. PRF was in the middle of the tube, between the red corpuscles at the bottom and acellular plasma at the top. **(B, C)** PRF was removed out of the tube by forceps and cut in the white horizontal circle. **(D, G)** Hematoxylin and eosin images of PRF showed two distinct zones, platelets and fibrin zones. Scale bars were 100 μm. **(E, F, H)** Scanning electron microscopy images of PRF showed fibrin fibers interconnected with each other and formed a three-dimensional, flexible, and elastic structure. Scale bars were 100 μm **(E)** and 50 μm **(F, H)**. **(I)** Transmission electron microscopy images of PRF showed that platelet aggregates were trapped in the fibrin fibers; some platelets were activated and degranulated. Scale bar was 1 μm. **(J–N)** ELISA quantifications of EGF, TGF-α, IGF-1, PDGF-BB, and VEGF-A released from PRF at 1, 3, 7, and 14 days, respectively. **(O)** Total amount of EGF, TGF-α, IGF-1, PDGF-BB, and VEGF-A released from PRF over 14 days. Error bars represent mean ± SE for *n* = 3.

Blood sample was separated into three layers after centrifugation. A fibrin clot was in the middle of the tube, between the red corpuscles at the bottom and acellular plasma at the top (**Figures 2A, B**). PRF had an elastic and tough smooth surface and a translucent texture (**Figure 2C**). HE staining showed two distinct zones in PRF, platelets and fibrin zone (**Figures 2D, G**). Platelets accumulated at the junction between the red corpuscles and the PRF clot, and the amount of blood cells was observed. Fibrin had a cross-linking mesh-like structure in pink color without platelets or any other cellular body in the upper part of the PRF clot. SEM showed that PRF had a large amount of fibrin fibers, which are long and narrow, interconnected to each other, and formed a three-dimensional, flexible, and elastic structure. Fibrin trigeminal structures can be seen everywhere in the porous network structure (**Figures 2E, F, H**). TEM of PRF showed that platelet aggregates were trapped in the fibrin fibers; each platelet was associated with several fibrin fibers. Some platelets were activated and degranulated, and form a slightly higher-density area (**Figure 2I**). PRF displayed a continual and steady release of growth factors over time (**Figures 2J-O**). The highest growth factor released from PRF was VEGF-A followed by PDGF-BB, EGF, TGF-α, and IGF-1. PRF released the highest amounts of IGF-1 on the first day, followed by a decrease in release at days 3, 7, and 14. EGF and VEGF-A released the maximum level at day 3. At day 7, PRF released the maximum level of TGF-α and PDGF-BB; PDGF-BB was not detected at day 14.

### 3.2 PRF exudates improve endometrial stromal cell proliferation and migration

**Figure 3:**
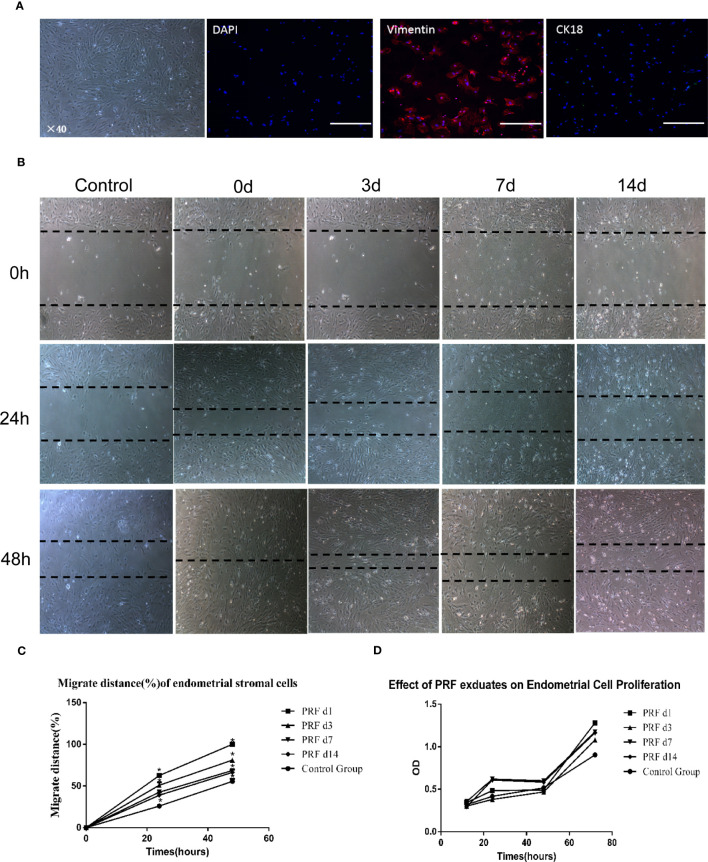
Effect of PRF exudates on rat endometrial stromal cells’ proliferation and migration *in vitro*. **(A)** Representative picture of rat endometrial stromal cells. Scale bar was 100 μm. Immunofluorescence microscopy confirmed that rat endometrial stromal cells were positive for vimentin (red color) and there was no expression of ck18 (green color). Scale bars were 500 μm. **(B, C)** Confluent monolayers of cells were scratched to form wound and cultured in the absence or presence of 20% PRF exudates at different time points: 0, 24, and 48 h. After 48 h, the cells in 1-day PRF exudates spread into the wound area; the migration rate of 3-day PRF exudates, 7-day PRF exudates, 14-day PRF exudates, and the control group was 81.1%, 68.52%, 66.3%, and 55.6%, respectively. Wound-healing assay showed that cell migration was significantly higher when cells were cultured with 20% PRF exudates. Scale bar was 100 μm (40×). **(D)** CCK8 assay showed that the proliferation level of cells in 1-day PRF exudates at 12 h, and 7-day PRF exudates at 24 and 48 h was significantly higher than that in the control group, *p* < 0.05. PRF exudates at other time points also promoted cell proliferation, but there was no statistical difference. *p < 0.05.

Phase-contrast photomicrographs illustrate the typical fusiform shape of stromal cells isolated from rat endometrial tissue, and cells were strongly positive for vimentin and negative for CK-18, showing no contamination with epithelial cells (**Figure 3A**). Wound-healing assay showed that cell migration was significantly higher when cells were cultured with 20% PRF exudates (**Figure 3B**). After 48 h, cells in 1-day PRF exudates spread into the wound area, and the migration rate of 3-day PRF exudates, 7-day PRF exudates, 14-day PRF exudates, and the control group was 81.1%, 68.52%, 66.3%, and 55.6%, respectively; 1-day PRF exudates had the most effect on promoting cell migration (**Figure 3C**). The CCK8 assay showed that the proliferation level of cells in 1-day PRF exudates at 12 h, and in 7-day PRF exudates at 24 and 48 h was significantly higher than that in the control group, *p* < 0.05. PRF exudates at other time points also promoted cell proliferation, but there was no statistical difference, *p* > 0.05 (**Figure 3D**).

### 3.3 Effect of the endometrium-damaged IUA model

**Figure 4:**
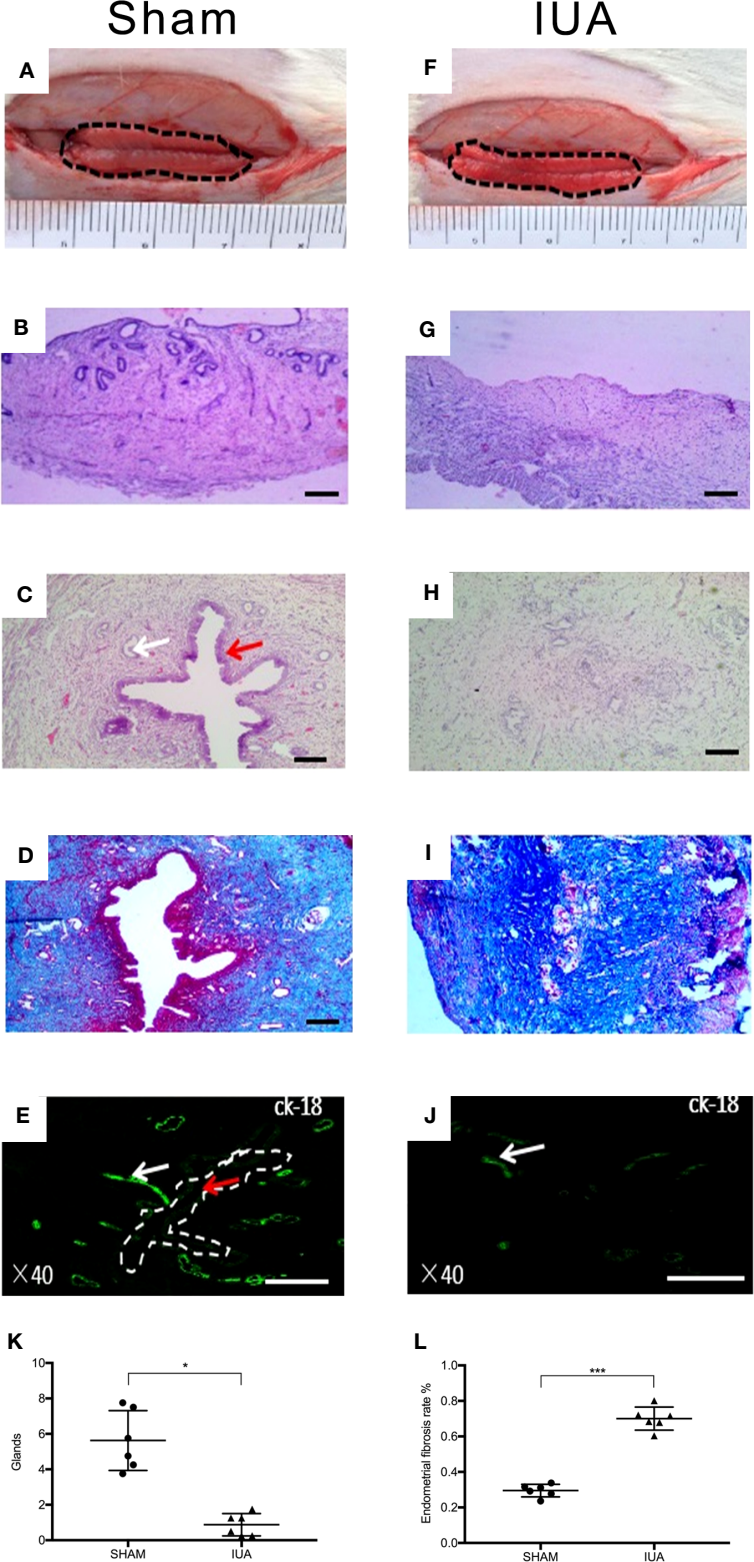
Establishment of a rat IUA model. **(A, F)** Gross observations of the inner surface of uterus before and after scraping. **(B, G)** Hematoxylin and eosin (HE) staining of uterus before and after scraping. **(C, H)** HE staining of the sham group uterus compared with the IUA group. After scraping for 14 days, uterus cavity and luminal epithelial cells were not observed and the number of endometrial glands was decreased. Red arrows and white arrows indicate endometrial epithelial cells in the luminal layer and endometrial glands, respectively. **(K)** Statistical results of number of endometrial glands in the sham and IUA group. Error bars were presented as mean ± SE for *n* = 6, *p < 0.05. **(D, I)** Masson staining of the sham group uterus compared with the IUA group. **(L)** The endometrial fibrosis rate of the IUA group was significantly higher than that of the sham group, ***p < 0.001. Scale bars of HE and Masson were 100 μm. **(E, J)** Immunofluorescence with antibody for cytokeratin-18 (CK-18), which stained endometrial epithelial cells. Rat endometrial luminal layer and endometrial glands were ck-18 positive, but only detected on endometrial glands in the IUA group. Scale bars were 500 μm.

A total of three 8-week-old female SD rats were used to identify the IUA model. The rats whose left horn was mechanically damaged were considered as the IUA group and those whose right horn had no damage were considered as the sham control. Before endometrium scraping, endometrial luminal surface was smooth with a normal folded structure (**Figure 4A**). HE staining showed that the normal uterus contained a uterine endometrial cavity, simple columnar epithelial cells on the surface, and endometrial glands in the endometrial stromal layer (**Figures 4B, C**). After endometrium scraping, endometrium became thinner, and a folded structure covered with coagulation and exudate was not observed (**Figure 4F**). HE staining showed that the endometrial epithelial layer was completely removed, and the stroma layer of the endometrium was also broken (**Figure 4G**). After 2 weeks, HE staining showed a normal structure of endometrium in the sham group (**Figure 4C**), collagen fibers were barely observed by Masson staining (**Figure 4D**), and CK-18-positive cells were distributed in the endometrial luminal layer and glands in the endometrial stromal layer (**Figure 4E**). However, in the IUA group, the uterus cavity and luminal epithelial cells were not observed and the number of endometrial glands was decreased significantly compared with the sham group (**Figures 4C, H, K**). Masson staining showed that the fibrotic area was significantly increased (**Figures 4I, L**). CK-18-positive cells were only found in the endometrial glands (**Figure 4J**). These results indicated a successful rat IUA model.

### 3.4 PRF improves damaged endometrium regeneration

**Figure 5:**
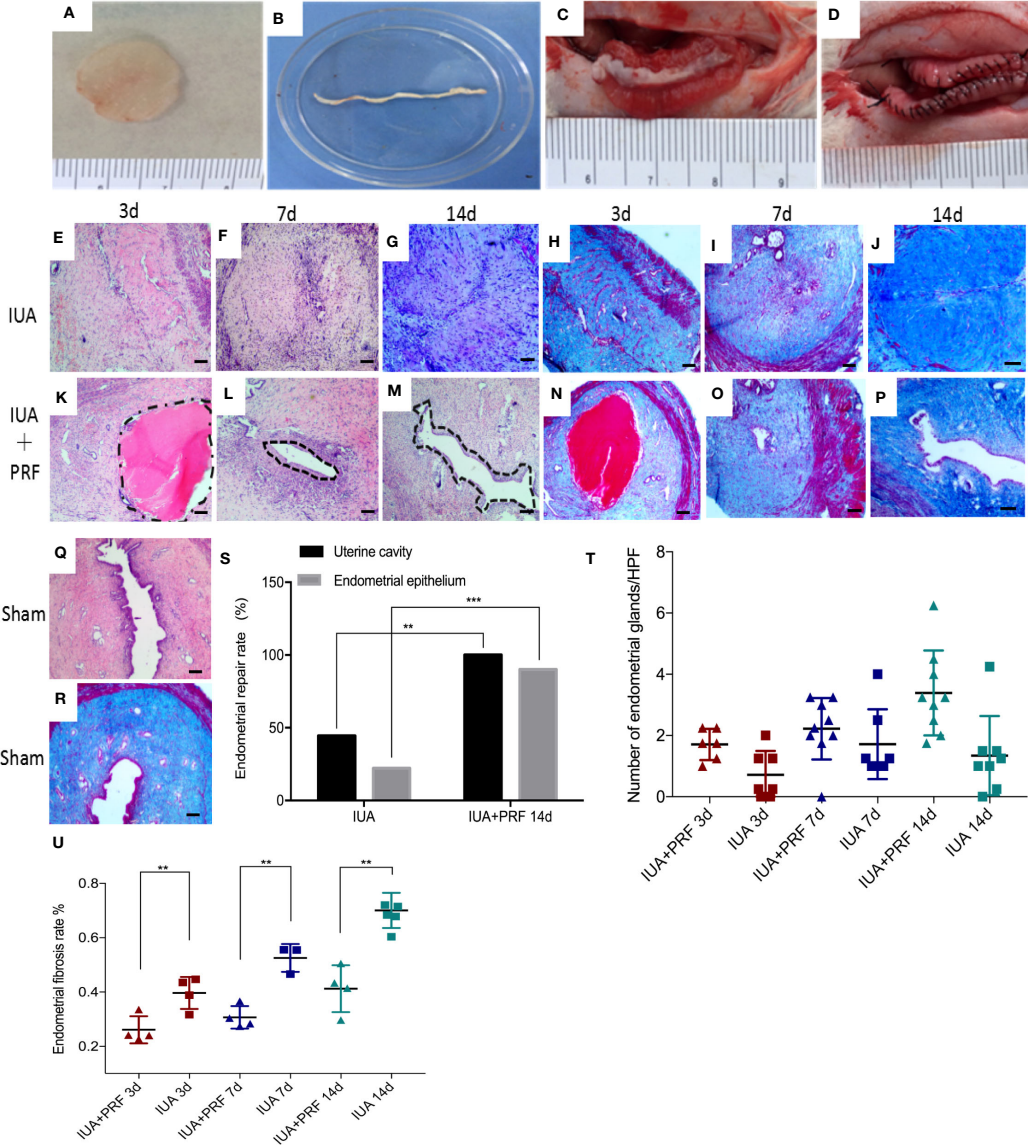
PRF promotes endometrial regeneration in the rat IUA model. **(A–D)** Preparation and transplantation of PRF to uterus after scraping. **(E–G)** and **(H–J)** show the uterine sectional specimens of the IUA group stained with HE and Masson at 3, 7, and 14 days. **(K–M)** and **(N–P)** show the uterine sectional specimens of the PRF transplantation group stained with HE and Masson at 3, 7, and 14 days. **(Q, R)** show the uterine sectional specimens of the sham group stained with HE and Masson. **(S)** Statistical results showed that PRF transplantation was beneficial for maintaining uterine structure and promoting endometrial luminal epithelium regeneration after transplantation of PRF for 14 days. **(T)** The number of endometrial glands was significantly higher than the IUA group after transplantation of PRF for 14 days. **(U)** The endometrial fibrosis rate of the PRF transplantation group was lower than that of the IUA group at 3, 7, and 14 days, respectively. Scale bars were 100 μm (×40). Error bars were presented as mean ± SE, *p < 0.05, **p < 0.01, ***p < 0.01.

PRF was gently pressed into a membrane with sterile dry gauze and cut into strips that are suitable for rat uterine cavity and then transplanted into the right horn after scraping the endometrium; the left horn only received curettage (**Figures 5A-D**). Three days after surgery, the uterine cavity and endometrial epithelial cells were not detected, and stromal layers completely adhered to each other in the IUA group (**Figure 5E**). In the PRF transplantation group, the uterine cavity existed and was occupied by homogeneous red dyed PRF but there were no epithelial-like cells in the center of the uterus (**Figure 5K**). Endometrial glands were all decreased compared to the sham group in both groups (**Figure 5Q**). Seven days after surgery, the IUA group showed no uterine cavity and stromal adhesion, which is the same as that at day 1 (**Figure 5F**). In the PRF transplantation group, PRF disappeared but endometrial cavity was observed (**Figure 5L**). Fourteen days after surgery, epithelial-like cells in the center of the uterus were found in the PRF transplantation group (**Figure 5M**). The IUA group showed the same structure as seen at days 3 and 7 (**Figure 5G**). The number of endometrial glands was significantly higher than the IUA group after transplantation of PRF for 14 days (**Figure 5T**). Masson staining showed that the endometrial fibrosis rate of the PRF transplantation group was lower than that of the IUA group at 3, 7, and 14 days, respectively (**Figures 5H-J, N-P, U**). Statistical results showed that PRF transplantation was beneficial for maintaining the uterine structure and promoting endometrial luminal epithelium regeneration after transplantation of PRF for 14 days (**Figure 5S**).

### 3.5 Evaluation of regenerative endometrium

**Figure 6:**
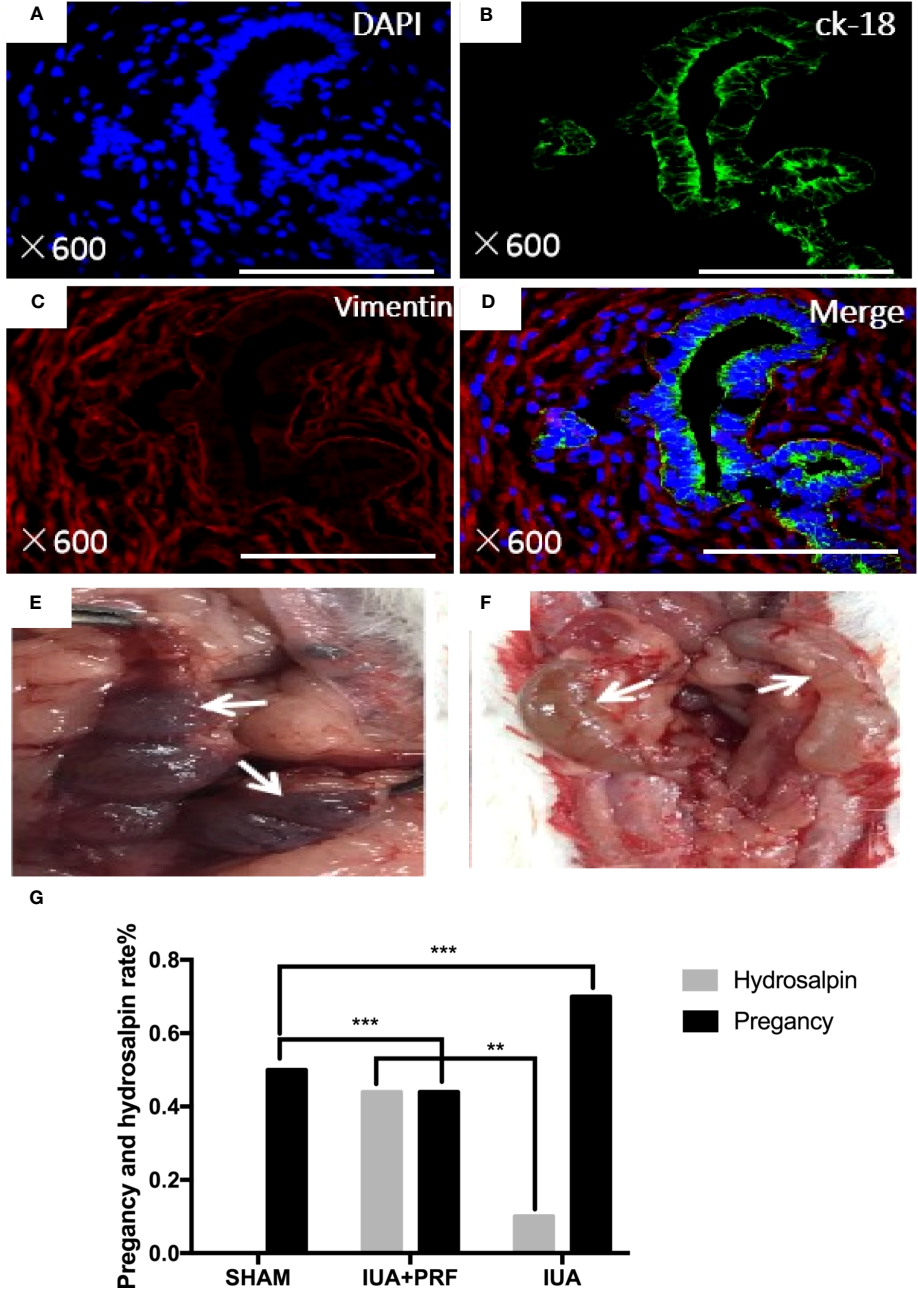
Identification of regenerated endometrium. **(A–D)** Immunofluorescence microscopy confirmed that regeneration endometrial luminal epithelial cells and endometrial glands were uniformly positive for ck-18 (green color). Endometrial stromal cells were positive for vimentin (red color). Scale bars were 500 μm. **(E)** After transplanting PRF for 6 months, pregnancy test showed that there were embryos that were implanted in the area where the endometrium was scraped (white arrow). **(F)** Hydrosalpinx in rat that was not pregnant (white arrow). **(G)** Statistical results showed that the PRF transplantation group had a high rate of pregnancy rate and a lower hydrosalpinx rate than the IUA group. **p < 0.01, ***p < 0.01.

Regeneration of endometrial luminal epithelial cells and endometrial glands was uniformly positive for CK-18, and ESCs were positive for vimentin (**Figures 6A-D**). Pregnancy test was used to evaluate the function of regenerative endometrium. A total of 24 rats were randomized into three groups, different from previous animal experiments; both horns were damaged in the experiment. After 30 days of recovery, they were mating with male adult rats 3:1, the number of fetus was calculated and all female rats were sacrificed for examination after 90 days. Live birth is only found In the sham group, the pregnancy rate was 50%(2/4). The average litter rate of pregnant rats was 7.5 pups/litter. Embryos in the uterus and hydrosalpinx were found when the abdominal cavity was opened in both the IUA and PRF transplantation groups (**Figures 6E, F**). Statistical results showed that the PRF transplantation group had a higher pregnancy rate (44.44%, 4/9 vs. 10%, 1/10) and a lower hydrosalpinx rate (44.44%, 4/9 vs. 70.00%, 7/10) than the IUA group (**Figure 6G**).

### 3.6 Endometrial stromal cell proliferation is a key factor for endometrium regeneration

**Figure 7:**
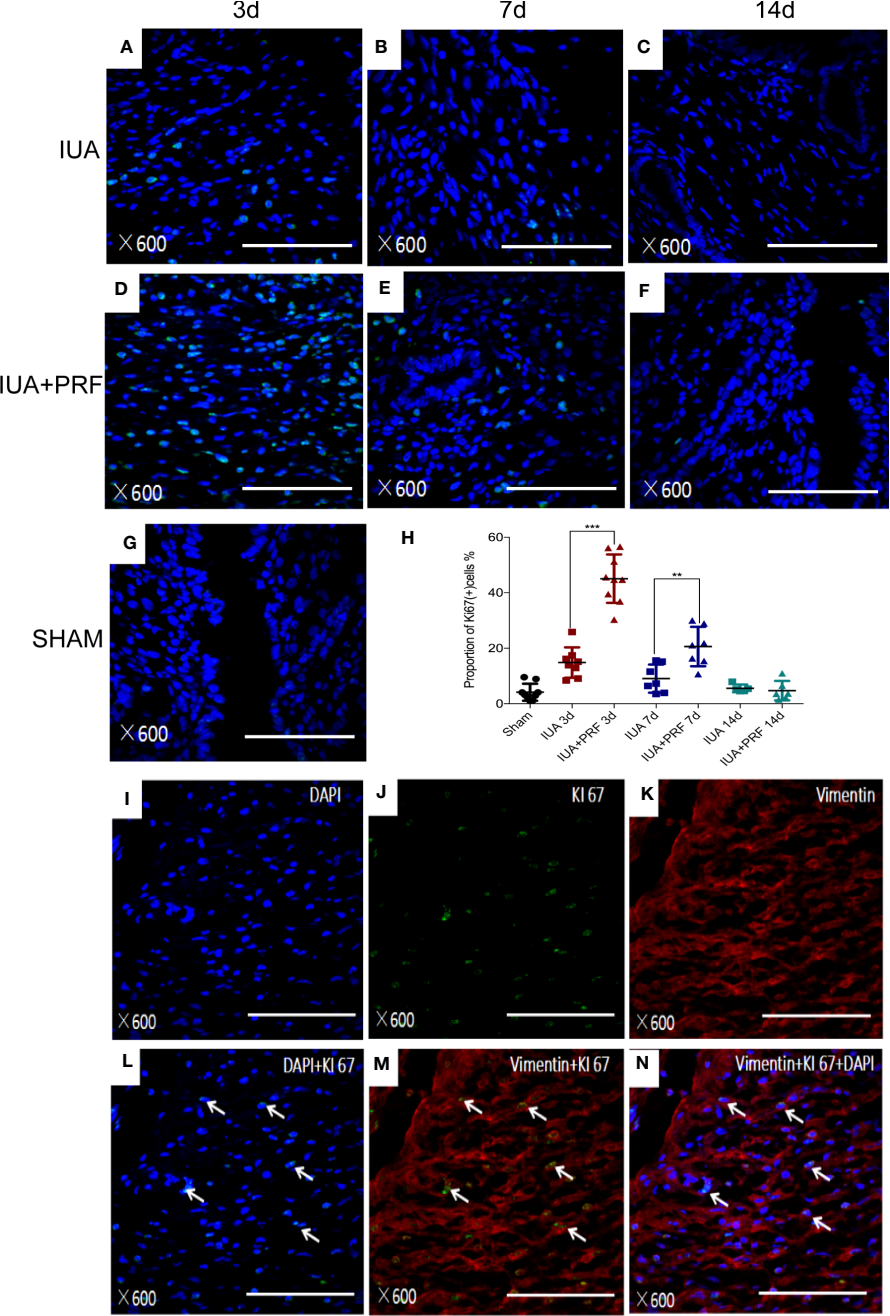
PRFs promote endometrial stromal cell proliferation *in vivo*. **(A–F)** Immunofluorescence microscopy showed the Ki-67(+) cells of the IUA group and the PRF transplantation group at 3, 7, and 14 days, respectively. **(G)** Immunofluorescence microscopy showed the Ki-67(+) cells of the sham group. **(H)** Statistical results showed that PRF transplantation could promote endometrial cell proliferation. The proportion of Ki-67(+) cells in the PRF transplantation group was significantly higher compared with the IUA group at 3 and 7 days. There was no statistical difference between groups at 14 days. Error bars were presented as mean ± SE, **p < 0.01, ***p < 0.01. **(I–N)** Immunofluorescence multiple staining showed Ki-67(+) cells (green color) also positive for vimentin (red color). Scale bars were 100 μm.

Immunofluorescence microscopy showed that Ki-67(+) cells had a maximum amount at day 3 and decreased by time (**Figures 7A-G**). Statistical results showed that PRF transplantation could promote endometrial cell proliferation. The proportion of Ki-67(+) cells in the PRF transplantation group was significantly higher compared with the IUA group at 3 and 7 days. There was no statistical difference between groups at 14 days (**Figure 7H**). We used immunofluorescence multiple staining to differentiate these cells from proliferating cells. Ki-67(+) cells were also shown to be positive for vimentin (**Figures 7I-N**).

## 4 Discussion

The results of this study suggest that PRF treatment was beneficial for damaged endometrium regeneration. To the best of our knowledge, this is the first study to evaluate the effect of PRF on damaged endometrium.

PRF is a second-generation autologous platelet concentrate; platelet-rich plasma (PRP) is reported as the first generation. Recent studies reported that PRP can promote endometrium regeneration *in vivo* and *in vitro* (13). Siwen Zhang showed that 10% of the activated PRP significantly promoted human menstrual blood-derived stromal cell proliferation and adipogenic/osteogenic dfferentiation while suppressing apoptosis (14), and improved the therapeutic effect of menstrual blood-derived stromal cells in a rat model of IUA (15). Lusine Aghajanova found that 5% of the activated PRP can promote the *in vitro* proliferation and migration of human endometrial stromal fibroblasts (eSFs), endometrial mesenchymal stem cells (eMSCs), and bone marrow-derived mesenchymal stem cells (BM-MSC) (16). Animal experiments show that autologous PRP stimulated and accelerated the regeneration of the endometrium and also decreased fibrosis in a murine model of damaged endometrium (17). Kim found that human PRP improved endometrial morphology, reduced degree of fibrosis, and downregulated the expression of fibrosis-related factors in a murine model of Asherman’s syndrome (18). In Ying Zhou’s study, it was reported that PRP therapy enhances the beneficial effect of bone marrow stem cell transplant on endometrial regeneration in a rat model (19). In Yajie Chang’s study, five patients with thin endometrium were treated with estrogen and 0.5–1 ml of PRP was infused into the uterine cavity on the 10th day of hormone replacement therapy (HRT) cycle. PRP was found to stimulate endometrial growth and five patients all had a successful pregnancy (20). Leila Nazari studied the therapeutic effect of PRP on patients with repeated implantation failure (RIF); 0.5 mL of platet-rich plasma was collected 48h before blastocyst transfer, and 18 of 20 participants were pregnant with 1 early miscarriage and 1 molar pregnancy (21). L. Aghajanova also presented two cases, which used PRP to treat Asherman’s syndrome, and had successful conception and ongoing clinical pregnancies with no short-term and long-term side effects (22). We found that PRF exudates promoted ESC proliferation and migration *in vitro* and PRF transplantation was beneficial for maintaining uterine structure and promoting endometrial luminal epithelium and endometrial gland regeneration *in vivo* in our study.

PRP was provided as venous blood is taken with anticoagulant, two steps of centrifugation, and artificial polymerization of the platelet concentrate (23). Several factors have been shown to limit the use of PRP: the preparation requires additional use of bovine thrombin or CaCl_2_, the liquid nature, and a very short release of growth factor profile (24). PRF derives from a natural and progressive polymerization occurring during centrifugation without anticoagulants. PRF retains a larger number of cytokines and growth factors in a supportive three-dimensional fibrin scaffold. Growth factors and cytokines are released continuously over a period of 10 days, and PRP has been shown to release the majority of its growth factors within the first day (9).

In our study, PRF displayed a continual and steady release of EGF, TGF-α, IGF-1, PDGF-BB, and VEGF-A over 14 days. These growth factors may aid in accelerating cell proliferation, angiogenesis, and cell migration, resulting in rapid healing and tissue regeneration. It is reported that TGF-α, EGF, and insulin-like growth factor-1 (IGF-1) participate in endometrial regeneration, mediating the mitogenic effects of estrogen and the differentiating effects of progesterone (25). EGF, TGF-β, and platelet-derived growth factor BB (PDGF-BB) have been investigated for their potential roles in supporting endometrial clonal forming unit (CFU) activity (26). Moreover, it is reported that PRF matrix enmeshes glycosaminoglycans (heparin and hyaluronic acid), which have a strong affinity with small circulating peptides and a great capacity to support cell migration and healing processes (27). The fibrin matrix stimulates the expression of integrin avb3, which allows cells to bind to fibrin (28). These characteristics of PRF suggest that PRF can act as a scaffold, facilitating endometrial cells’ migration. In addition, it is worth mentioning that we found a lower degree of neutrophil infiltration in the PRF group compared to the IUA group at 3, 7, and 14 days after transplantation (**Supplementary Figure 1**). Studies have shown that activated neutrophils may release neutrophil extracellular traps (NETs) during a distinct form of cell death; NETs are rich in bioactive molecules that promote fibrosis (29). This suggests that PRF may play an anti-fibrotic role by attenuating the infiltration of neutrophils.

Platelets are 1- to 4-μm-sized anucleate cells that break off from megakaryocytes. During the wound-healing process, platelets are activated and release amounts of cytokines that can promote re-epithelialization and angiogenesis. It is reported that platelets play a role not only in wound healing, but also in atherosclerosis, liver regeneration (30), and hypoxia-induced angiogenesis (31). Koh Suginami reported that platelets promote re-epithelialization during menstruation and found that extravasated platelets were deposited among the endometrial stroma just beneath the regenerating endometrial luminal surface during the menstrual period. When a human endometrial epithelial cell-derived immortalized cell line (EM-E6/E7/hTERT cells) is co-cultured with platelets, platelets promoted the cell to-Matrigel attachment of EM-E6/E7/hTERT cells and also promoted cell-to-cell contact among EM-E6/E7/hTERT cells in parallel with E-cadherin expression (32), and they demonstrated that platelet-derived soluble factors and microparticles released by platelets may be involved in the process of endometrial re-epithelialization in their later study (33). The platelet content of PRF is four to five times higher than that in normal blood, and platelets in PRF may play an important role in the process of endometrial epithelialization.

Immunofluorescence microscopy showed that Ki-67(+) cells in the PRF transplantation group were significantly higher compared with the IUA group at day 3 and day 7. Proliferating cells were also positive for vimentin, which is the marker for stroma. It suggests that ESCs may be a key factor for endometrium regeneration. The mechanism of endometrium regeneration, including re-epithelialization, is controversial; a previous study proved re-epithelialization of the luminal epithelium by proliferation of residual glandular stumps (34, 35), and recent studies demonstrated that a proportion of stromal cells differentiate into both luminal and glandular epithelial cells after parturition and contribute to the re-epithelialization process (36). R Garry’s study found that in early endometrial epithelial repair after menstruation, dead epithelial cells are shed from the glands and replaced by new small epithelial cells that appear to arise by differentiation of the surrounding stromal cells, which may be endometrial progenitor/stem cells (37).

Histological analysis confirmed that regeneration of endometrial luminal epithelial cells and endometrial glands was uniformly positive for CK-18, and ESCs were positive for vimentin. It suggests that endometrial regeneration has a normal histological structure. Pregnancy test showed that live birth was only found in the sham group and the pregnancy rate was only 50%, suggesting that incision and suture itself may affect pregnancy outcome. The pregnancy rate in the PRF transplantation group is lower than that in the sham group but higher than that in the IUA group. It is reported that PRF has anti-inflammatory potential (38). PRF transplantation could decrease hydrosalpinx rate *via* reducing inflammation so that pregnancy rate would increase. Multiple studies have demonstrated high rates of infertility and pregnancy complications in patients with IUA even after surgical repair (39), because of the poor endometrial receptivity (40). Whether PRF can improve endometrial receptivity requires further study.

The prevalence of IUA ranges from 16% to 24% in women undergoing pregnancy-related curettage (41). It is reported that all kinds of hysteroscopic procedures will cause IUA, such as hysteroscopic myomectomy, hysteroscopic septum resection, and hysteroscopic polypectomy. Hysteroscopic myomectomy has the highest prevalence, ranging from 31% to 45% (42). Our data demonstrated that intrauterine administration of PRF was effective in preventing IUA and stimulated damaged endometrium regeneration in rats. PRF provided a promising method for women with poor endometrium. The value of PRF in the field of gynecology deserves further research.

Although PRF has shown better results in rats with uterine adhesions caused by mechanical injury, it is not necessarily indicated for the treatment of all types of uterine adhesions. A study by Stewart et al. showed that thrombosis was shown when proliferation of endometrial cells is strongly altered (1). This suggests that future studies of PRF for uterine adhesions should focus on populations with a high propensity for endometrial thrombosis. The present study has many limitations.

This study refers to clinical experience, and the control group is only subjected to surgical curettage, without PRF analogues as a more rigorous control of the experiment. Owing to the limited experimental time, this study did not follow up the birth of the pups of the experimental group animals. There should be more studies on the effects of PRF on animal litter and reproductive system in the future. In addition, the mechanism of PRF in uterine adhesions should be investigated in more depth in the future in order to provide a basis for the clinical application of PRF.

## 5 Conclusions

Intrauterine administration of PRF was demonstrated to be effective in preventing IUA and stimulated damaged endometrium regeneration in rats. This study not only provided a promising method for its potential in endometrium regeneration in women who suffer from uterine infertility but also may prevent adhesion after intrauterine surgery in clinical cases.

## Data availability statement

The original contributions presented in the study are included in the article/**Supplementary Material**. Further inquiries can be directed to the corresponding author.

## Ethics statement

The animal study was reviewed and approved by Ethics Committee of Beijing Shijitan Hospital, Capital Medical University.

## Author contributions

All authors contributed to the study conception and design. Methodology was performed by LM, XW, and YS. Formal analysis was performed by MY and XC. Original draft was written by LM. Writing—review and editing were performed by LM and XW. Supervision was performed by LC and WB. All authors contributed to the article and approved the submitted version.

## Supplementary material

Supplementary Figure 1
**(A)** Neutrophil infiltration at different time points in the PRF and IUA groups. Scale bars were 25 μm. **(B)** Histogram plot of neutrophil count in the PRF group vs. the IUA group.
