# Supplementary figures and images for: Platelet-rich fibrin improves repair and regeneration of damaged endometrium in rats
Source: Front Endocrinol (Lausanne). 2023 Aug 8;14:1154958. doi: 10.3389/fendo.2023.1154958 (PMC10443704; doi:10.3389/fendo.2023.1154958)

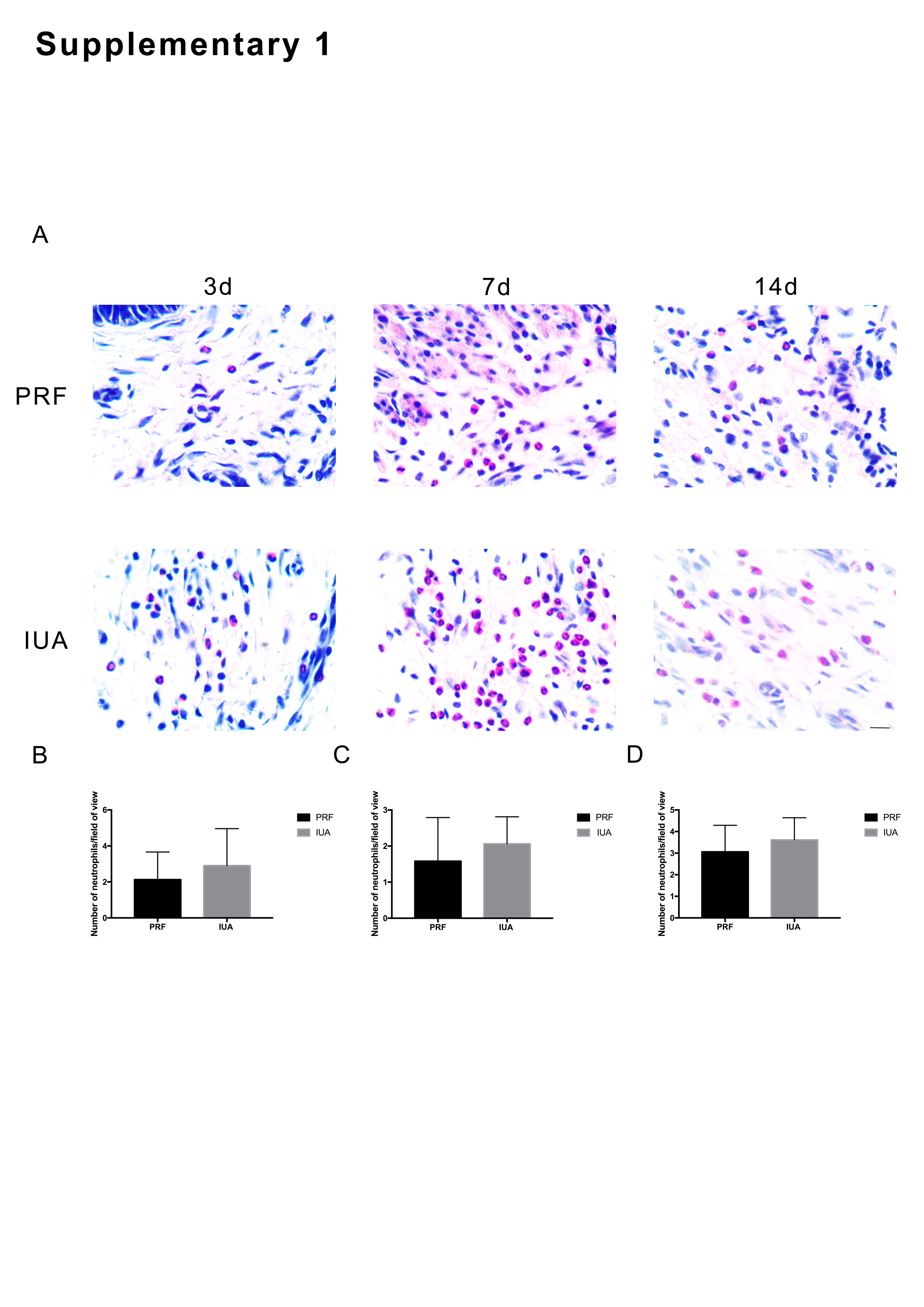

Supplement: Supplementary Figure 1 — (A) Neutrophil infiltration at different time points in the PRF and IUA groups. Scale bars were 25 μm. (B) Histogram plot of neutrophil count in the PRF group vs. the IUA group. [file Image_1.jpeg]
